# The Presence of an Anterior Tubercle in the Foramen Magnum of the Cranium: A Case Report With Potential Clinical Consequences

**DOI:** 10.7759/cureus.96187

**Published:** 2025-11-06

**Authors:** George K Paraskevas, Alexandros Poutoglidis, Christos Lyrtzis, Chrysanthos Chrysanthou, Nektarios Galanis, Xanthippi Partalidou

**Affiliations:** 1 Department of Anatomy and Surgical Anatomy, School of Medicine, Aristotle University of Thessaloniki, Thessaloniki, GRC

**Keywords:** anatomy & physiology, ent procedures, neurosurgery, posterior fossa, skull base pathologies

## Abstract

There are not so many variants regarding the foramen magnum (FM). However, the existence of a prominent eminence at the anterior border of FM is one of the variations. In the current study, we detected such a tubercle in a dried skull of an old man's skeleton, with its base arising from the midpoint of the internal aspect of the anterior margin of the FM. The apex of this tubercle was oriented backwards. As for the dimensions, the anteroposterior distance was measured at 4 mm, and the transverse distance at 5 mm. That tubercle is usually circular or ovoid in shape, and its appearance is commonly overlooked by radiologists and physicians. That eminence can potentially damage the pyramidal fibers of the spinal cord in extreme flexion of the head, usually during a cranial trauma. Both radiologists and clinicians should be aware of this rare variant, as it may be an incidental radiographic finding or a reason for damage to the spinal cord during head and neck injuries. Moreover, there is a positive cost-effectiveness for the patient and the public health system of their country, since the costs for both will be eliminated if the patient takes their precautions.

## Introduction

The skull base is one of the most complex anatomical regions in the human body. It separates the cranial cavity from other structures and consists of five bones. These are the ethmoid, sphenoid, temporal, frontal, and occipital bones. The skull base is subdivided into three fossae: the anterior, the middle, and the posterior [1].

The posterior fossa lies in the most inferior part of the skull base and hosts the cerebellum, pons, and medulla oblongata. Part of this fossa is the foramen magnum (FM). The borders of this foramen are the occipital condyles laterally, the basiocciput anteriorly, and the supraocciput posteriorly.

The FM divides the brain from the spinal cord. In some mammals, the FM can be located anteriorly [2]. The diameter differs between genders, with men tending to have larger diameters; in the presence of the tubercle, the overall diameter decreases. The FM's shape can vary from circular to ovoid and, in rare cases, to rhomboid or hexagonal [1].

The FM consists of a big opening in the skull base. The medulla oblongata passes through this and continues its course as the spinal cord. The borders of the FM are the basiocciput anteriorly, the supraocciput posteriorly, and the occipital condyles laterally. The anterior part of the FM may demonstrate significant anatomical variations, such as the presence of an anterior tubercle [1].

In the present study, we describe a rare case of an anterior tubercle located at the anterior margin of the FM. We discuss its anatomical characteristics, relevant literature, and potential clinical implications that may arise when such an uncommon variant is overlooked in radiological or clinical practice. Herein, we present a rare case of an anterior tubercle at the FM of a skull, discuss its relevant literature and potential clinical significance, and address the socioeconomic issues raised when a radiologist or other physician overlooks such a rare variant.

## Case presentation

**Figure 1:**
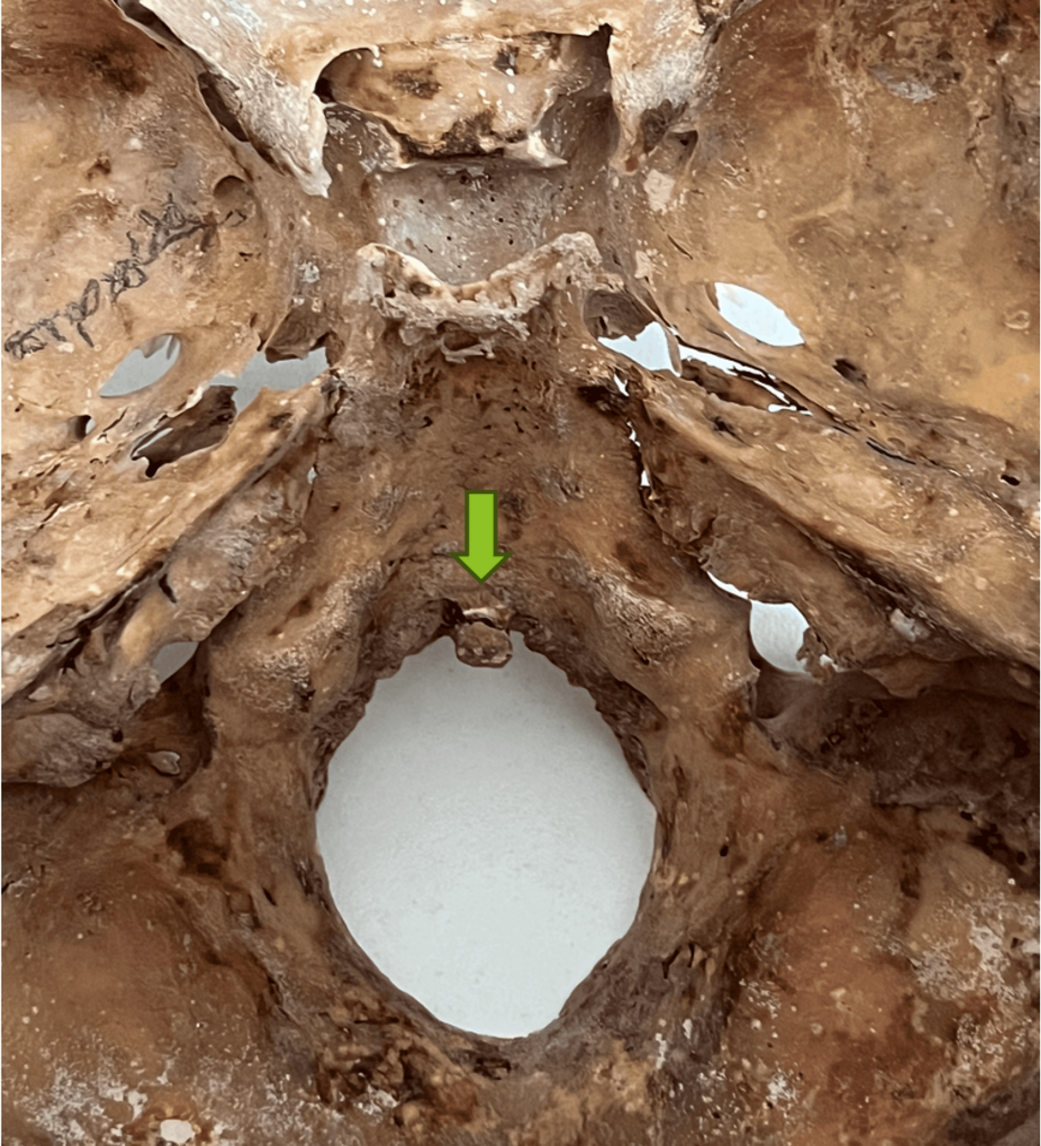
Presence of an anterior tubercle in the anterior part of the foramen magnum. The arrow indicates its location.

During the course of routine screening of human bones, a dried skull belonging to a White man, who died at the age of 82, was observed in the Laboratory of Anatomy and Surgical Anatomy in the Aristotle University of Thessaloniki. The complete skeleton was donated to the laboratory for educational and research purposes. A very rare anatomical variation was discovered in the FM. Specifically, an anterior tubercle was observed at the midpoint of the internal aspect of the anterior margin of the FM, projecting posteriorly into the cranial cavity. The apex of this tubercle was oriented backwards. The texture of the anterior tubercle was very smooth in continuity with the occipital bone. There was no evidence of remodeling, fracture, or any other degenerative process. Therefore, our assumption is that the tubercles were congenital or even developmental. The dimensions of the tubercle were measured with the application of a digital Vernier caliper. As for the dimensions, the anteroposterior distance was measured at 4 mm, and the transverse distance at 5 mm (Figure 1). The overall anteroposterior diameter of the FM was 34 mm (decreased by 4 mm because of the presence of the tubercle), and the transverse diameter was 32 mm. No other abnormalities were observed in this skeleton.

## Discussion

The anterior tubercle of the foramen magnum (ATFM) has presumably been described first by LeDouble in 1903, who compared that structure with the medial condyles of birds and reptiles [3]. The presence of an ATFM has clinical implications, as it can cause compression to the structures that pass through the FM [4]. In addition, some authors have described periodontal ligament degeneration associated with basilar invagination secondary to atlanto-axial joint osteoarthritis [4,5].

In 1991, Lakhtakia et al. presented a large series of 422 skulls to record the presence of the tubercle. An ATFM was present in 15% of cases, and its shape and dimensions varied significantly among skulls. The ATFM varied in length from 0.5 to 4 mm. The same authors hypothesized that such an anterior tubercle can damage the pyramidal fibers of the spinal cord during extreme head flexion [6]. Ahmed et al. [7] found the length and the width of the ATFM to be more than double compared with the measurements of Lakhtakia et al. [6].

In 1996, Vazquez et al. presented a case series of 382 skulls and found an incidence of 1.3%. They considered that this tubercle is derived from the ossification of the apical ligament of the dens. Furthermore, they concluded that due to the proximity of the anterior tubercle to the spinal cord, the latter could be damaged by it in cases of cranial trauma [8].

In 2019, Aragão et al. published a case series of 350 FM and found an incidence of only 0.28% [9]. They concluded that both radiologists and clinicians should be aware of this entity, as it may be an incidental radiographic finding or a cause of compressive phenomena in cranial structures.

The presence of the ATFM may have significant clinical implications. A prominent foramen can cause compression to the adjacent neurovascular structure, including the medulla oblongata and the vertebral arteries. Movements such as neck flexion can cause pain. Medullary compression may be associated with headaches, neck stiffness, limb weakness, or even dizziness. In addition, the anterior tubercle may cause diagnostic problems, mimicking fracture fragments or pathological calcifications.

The integration of early detection interventions and patient navigation programs has consistently demonstrated the potential to reduce overall health expenditures and improve resource efficiency, primarily by avoiding costly late-stage treatments [10,11].

## Conclusions

Awareness of such anatomical variation and its early diagnosis is crucial for physicians, as they can help protect individuals with ATFM by informing them to avoid extreme head and neck motions, especially during specific athletic activities, thereby helping them protect the spinal cord from cranial and neck trauma. Moreover, there is a positive cost-effectiveness for the patient and the public health system of their country, since the costs for both will be eliminated if the patient takes their precautions.

Awareness should be raised among radiologists to promptly identify the presence of ATFM. This will have an impact on minimizing the risk of misdiagnosis. Our results should be verified by future studies to determine the exact effects of ATFM on the health system.
